# Authors’ response: Sequencing bias for residue 28 of the neuraminidase of the recent highly pathogenic avian influenza virus A(H5N8)

**DOI:** 10.2807/1560-7917.ES.2021.26.36.2100871

**Published:** 2021-09-09

**Authors:** Ivan M Susloparov, Natalia P Kolosova, Aleksey V Danilenko, Aleksandr B Ryzhikov

**Affiliations:** 1 State Research Centre of Virology and Biotechnology “Vector” Rospotrebnadzor, Koltsovo, Novosibirsk Region, Russia

**Keywords:** avian influenza, H5N8, S28N, neuraminidase, human influenza, influenza adaptation

**To the editor:** We concur with the Wen et al. that currently, the S28N amino acid substitution in the neuraminidase (NA) of influenza A(H5N8) viruses is rare [1]. This conclusion was based on the observation of the authors that the amino acid substitution is only found in 0.39% of the influenza A(H5N8) strains deposited in GISAID by 28 August 2021, which is also mentioned in our paper [2].

The A/Astrakhan/3212/2020 MDCK isolate had the S28N substitution (coverage 2,155 sequences per codon, 98.5% AAC – amino acid N). We appreciate the authors’ investigation into the question of the origin and selection of the S28N mutation in the isolate that was obtained from the primary material there the mutation was not detected. We agree that one of the possible explanations proposed in our paper as nested PCR bias does not cover all possible scenarios.

The origin of the S28N mutation in the virus cultivar could be explained by several hypotheses. One of the hypotheses presented by Wen et al. is that the mutation was absent in the primary material and was acquired as a result of virus adaptation to Madin-Darby canine kidney (MDCK) cells. Another hypothesis could be that the virus variant with the mutation was already present in the clinical specimen and was selected for in MDCK sells.

We performed a more thorough analysis of next generation sequencing (NGS) data for the presence of single nucleotide polymorphisms as described in Danilenko et al. [3]. This analysis revealed that the primary material contained a virus variant with the mutation S28N with a frequency of 0.17% (AGC codon (amino acid S) – 224,955 reads per codon and AAC codon (amino acid N) – 373 reads per codon). We previously demonstrated that the frequency for the AAC codon is at the limit of detection at this NGS coverage [3]. Presence of the S28N mutation in the clinical sample may also be supported by the detection of the mutation in one of the avian isolates A/chicken/Astrakhan/321–06/2020 from the outbreak (which was propagated in chicken eggs), which is indicative of possible human exposure to the virus variant during the outbreak. Thus, it is possible that the virus with the S28N mutation was present in the clinical material in a barely detectable minor quantity and was selected for in MDCK cells. However, that would require a very strong selection given the complete dominance of the viral variant with the S28N mutation in a second MDCK passage. Analysis of GISAID data showed that from all entries with reported virus source, 28N was reported only in viruses from two original specimens, one cell isolate and one egg isolate. 28S in NA was reported in 395 influenza A(H5N8) viruses sequenced from primary material, in 71 viruses sequenced from cell culture (including MDCK) and in 441 viruses cultivated in eggs (GISAID data by 6 September 2021). Rare detection of the S28N mutation in cell isolates (one in 72) does not support the hypothesis of strong selection for the S28N mutation in MDCK cells. However, cultivation of A/Astrakhan/3212/2020 and the presence of 28N in H5N6, H5N1 and H7N9 viruses that infect humans [2] suggest a possibility that the S28N mutation may provide a certain advantage in the selection of the virus variant in mammalian cells. This hypothesis needs to be studied further.

Another hypothesis would be that viable virus in the clinical specimen that was successfully propagated in MDCK contained the S28N substitution because of low-level replication in the nasal cavity, while for the virus variant with 28S, no viable virus was present and mostly genetic material was remaining at the time the clinical specimen was taken.

Amino acid substitutions occurring in the transmembrane and stalk region of NA (around position 27 in influenza A(H3N2) viruses) during passaging on MDCK and other mammalian cell cultures have previously been reported, and it has been suggested that they may contribute to increased stability or budding efficiency of the influenza viruses in cell culture [4].

As we noted in the paper: “*Currently, no information on the phenotypic significance of the mutation is available*”. Investigation of the possible role of the S28N amino acid substitution in virus selection and propagation in MDCK cells and in animal models (e.g. using reverse genetics) may lead to a better understanding of the role of the mutation in influenza A(H5N8) virology and human infection.

## References

[1] Guo J Wang C Huang S Wen F. Letter to the editor: Sequencing bias for residue 28 of the neuraminidase of the recent highly pathogenic avian influenza virus A(H5N8). Euro Surveill. 2021;26(36):2100819. 10.2807/1560-7917.ES.2021.26.36.2100829 PMC8431995 34505566

[2] Pyankova OG Susloparov IM Moiseeva AA Kolosova NP Onkhonova GS Danilenko AV Isolation of clade 2.3.4.4b A(H5N8), a highly pathogenic avian influenza virus, from a worker during an outbreak on a poultry farm, Russia, December 2020. Euro Surveill. 2021;26(24):2100439. 10.2807/1560-7917.ES.2021.26.24.2100439 34142650 PMC8212591

[3] Danilenko AV Kolosova NP Shvalov AN Ilyicheva TN Svyatchenko SV Durymanov AG Evaluation of HA-D222G/N polymorphism using targeted NGS analysis in A(H1N1)pdm09 influenza virus in Russia in 2018-2019. PLoS One. 2021;16(4):e0251019. 10.1371/journal.pone.0251019 33914831 PMC8084186

[4] Barnard KN, Wasik BR, Alford-Lawrence BK, Hayward JJ, Weichert WS, Voorhees IEH, Holmes EC, Parrish CR. Influenza A viruses serially passaged in different MDCK cell lines exhibit limited sequence variation across their genomes, with the exception of the hemagglutinin gene. bioRxiv 2020.02.20.959015. https://doi.org/10.1101/2020.02.20.959015

